# Tissue factor overexpression promotes resistance to KRAS-G12C inhibition in non-small cell lung cancer

**DOI:** 10.1038/s41388-023-02924-y

**Published:** 2024-01-08

**Authors:** Yu Zhang, Liang Liu, Jinpeng Pei, Zhiqiang Ren, Yan Deng, Ker Yu

**Affiliations:** Department of Pharmacology, Fudan University School of Pharmacy, Shanghai, China

**Keywords:** Non-small-cell lung cancer, Tumour immunology

## Abstract

The recently approved KRAS^G12C^ mutation-specific inhibitors sotorasib and adagrasib (KRAS^G12C^-I) represent a promising therapy for KRAS^G12C^-driven non-small cell lung cancer (NSCLC). However, many eligible patients do not benefit due to intrinsic or acquired drug resistance. Tissue factor (TF) is overexpressed in KRAS-mutated (KRASmut) NSCLC and is the target of the FDA-approved ADC Tivdak. Here, we employed HuSC1-39, the parent antibody of a clinical stage TF-ADC (NCT04843709), to investigate the role of TF in KRASmut NSCLC. We found that patients with TF-overexpression had poor survival, elevated P-ERK/P-AKT activity levels and low immune effector cell infiltration in the tumor. In a panel of KRAS^G12C^ cell lines, KRAS^G12C^-I response correlated with suppression of TF mRNA, which was not observed in resistant cells. In the drug resistant cells, TF-overexpression relied on an mTORC2-mediated and proteasome-dependent pathway. Combination treatment of HuSC1-39 or mTORC1/2 inhibitor MTI-31 with KRAS^G12C^-I each produced synergistic antitumor efficacy in cell culture and in an orthotopic lung tumor model. TF-depletion in the resistant cells diminished epithelial mesenchymal transition, reduced tumor growth and greatly sensitized KRAS^G12C^-I response. Moreover, employing immunohistochemistry and coculture studies, we demonstrated that HuSC1-39 or MTI-31 reset the tumor microenvironment and restore KRAS^G12C^-I sensitivity by reshaping an M1-like macrophage profile with greatly enhanced phagocytic capacity toward tumor cell killing. Thus, we have identified the TF/mTORC2 axis as a critical new mechanism for triggering immunosuppression and KRAS^G12C^-I resistance. We propose that targeting this axis with HuSC1-39 or MTI-31 will improve KRAS^G12C^-I response in KRAS-driven NSCLC.

## Introduction

KRAS is one of the most prevalent oncogenic drivers of human malignancy including lung cancer. Approximately 25% of NSCLC harbor the mutated KRAS gene (KRASmut), among which KRAS^G12C^ is the most common type in lung adenocarcinoma (LUAD) [1, 2]. Oncogenic KRASmut acts as constitutively activated GTP-bound state, leads to activation of downstream mitogen-activated protein kinase (MAPK/ERK) and phosphatidylinositol 3-kinase (PI3K) signaling pathways [3, 4]. In lung cancer, the recently approved KRAS^G12C^ mutation-specific inhibitors sotorasib (AMG510) and adagrasib (MRTX849), referred to as KRAS^G12C^-I herein, have shown improved clinical efficacy compared to standard chemotherapy [5]. However, among the eligible patients the overall response rate remains low and response duration to AMG510 is considered unsatisfactory [6]. Similarly, patients treated with MRTX849 initially had an objective response to treatment, followed by disease progression with remained activation of the RTK-RAS signaling pathway [7]. Moreover, many resistant tumors showed significant activation of the AKT/mTOR signaling pathways and downregulated multiple immune signals associated with activation of antitumor immunity, suggesting that immune escape could be a critical feature of acquired resistance to KRAS inhibitors [8, 9]. Therefore, new therapeutic strategies are required to delay and overcome KRAS resistance in cancer patients.

Tissue factor (TF), also known as coagulation factor III, is a transmembrane single-chain glycoprotein with 263 amino acid residues. The physiological role of TF is to initiate extrinsic coagulation cascade when the tissue is injured [10]. Although TF is critical for normal hemostatic functions, its activation can greatly promote the malignant behavior of cancer. TF is abnormally elevated on the surface of many types of malignant tumor cells and vascular endothelial cells, including those of lung cancer, triple-negative breast cancer (TNBC), pancreatic cancer, glioma, acute lymphoblastic leukaemia and others [11–14]. Numerous reports have shown that TF participates in tumor growth, angiogenesis and epithelial-mesenchymal transition (EMT) [15–18]. Induction of an EMT-like phenotype could increase TF expression in epithelial cancer cells that are resistant to radiation therapy, chemotherapy, and targeted therapy [19]. The TF-FVII complex binds and activates intracellular protease-activated receptor 2 (PAR2) signaling, which in turn stimulates MAPK and PI3K signaling pathways [20, 21]. The TF-PAR2-mediated intracellular signaling may operate in a parallel or bypass mechanism in supporting tumor growth and therapy resistance. However, whether TF is frequently overexpressed in KRASmut NSCLC and whether it plays a functional role in KRAS inhibitor resistance remains unknown.

Tumor microenvironment (TME) plays an increasingly important role in therapy outcome. In many settings, therapy resistance is caused by the tumor cell and the interaction between the tumor and TME [22]. TME contains multiple immune cells, of which TAMs are the most abundant in almost all malignancies [23]. TAMs are typically M2-polarized cells, they do not promote immune responses to tumor cells. Instead, they exhibit the oncogenic properties of primary cancer, including supporting immunosuppression, metastasis, disease progression and therapy failure [24, 25]. In contrast, the M1-like macrophages have pro-inflammatory and tumoricidal effects, often reflecting a favorable treatment outcome [26, 27]. Tumor-mediated immune escape could be facilitated by suppressing macrophage phagocytosis in the TME [28, 29]. We are interested in learning whether TF regulates intracellular signals to favor a protumor TME. In particular, whether TF modulates macrophage recruitment, polarization and/or phagocytotic capacity in TME remains largely unknown.

TF is the target of the recently FDA-approved ADC Tivdak. In this study, we employed HuSC1-39 [30], the parent antibody of a clinical stage TF-ADC (NCT04843709), to investigate the functional role of TF in KRASmut NSCLC. We performed database analysis, tumor tissue microarrays (TMA) stanning, in vitro and in vivo studies to identify TF as an important player in tumor immune escape and drug resistance in KRASmut NSCLC.

## Results

### Tissue factor (TF) overexpression correlates with tumor progression and immunosuppressive tumor microenvironment in KRASmut LUAD

**Fig. 1:**
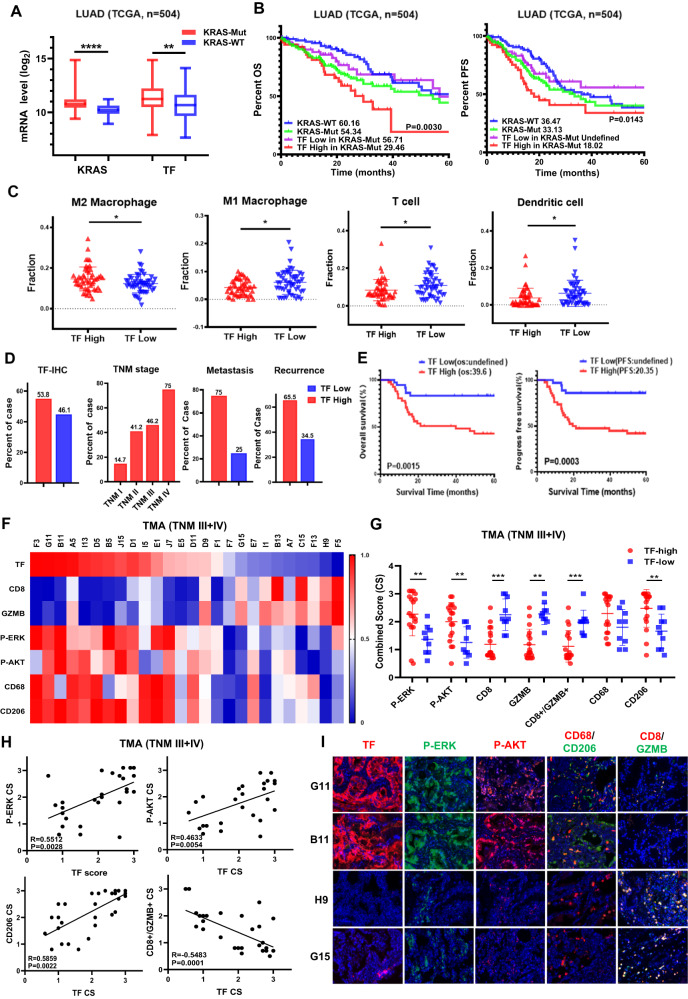
TF is elevated in KRASmut NSCLC and correlates with poor survival and immunosuppressive TME. **A** TCGA datasets were analyzed for TF and KRAS mRNA expression. **B** OS and PFS of TF high and TF low groups were calculated by Kaplan-Meier method. **C** The infiltration levels of immune cells in TF high vs TF low groups in TCGA cohort. **D** KRASmut lung cancer tissue microarrays were subjected to IHC staining. TF expression was analyzed versus the indicated clinical parameters. **E** OS and PFS in negative/low (*n* = 36) and moderate/high (*n* = 42) TF expression groups were calculated by Kaplan-Meier method. **F** IHC was performed for the indicated biomarkers, quantified as described in methods and plotted as expression level heatmap in KRASmut LUAD patients of stage III and IV. **G** The levels of P-ERK, P-AKT and immune cell markers in TF high/low KRASmut LUAD patients of stage III and IV (*n* = 29). **H** Comparing the correlation between P-ERK, P-AKT, CD206, CD8A^+^GZMB^+^ and TF expression in KRASmut LUAD patients of stage III and IV. **I** IHC profile of representative TF-high/low tumors are shown. Loss of tissue during staining was not included in the analysis. *P < 0.05, **P < 0.01, ***P < 0.001.

KRASmut occurs frequently in lung adenocarcinoma (LUAD). We therefore analyzed TCGA LUAD RNA-seq database. Tumors with KRASmut tended to express higher levels of TF mRNA compared with that of KRAS wild type (KRASwt) tumors (Fig. 1A). Elevated TF mRNA was also observed in KRASmut lung cancer with STK11 and KEAP1 co-mutations, but there was no significant difference with TP53 co-mutations (Fig. S1A).

Survival analysis showed significantly lower OS and PFS in patients with KRASmut tumors and TF-high expression (Fig. 1B). As TNM stage classification denotes an overall degree of malignancy reflecting tumor growth and metastasis, we performed additional TNM stage-matched analysis and observed a similar tendency (Fig. S1B, S1C). As tumor environment influences therapy outcome, we analyzed tumor associated immune cell profile employing CIBERSORT algorithm. TF mRNA levels were negatively correlated with several immune cell infiltrates including M1-like macrophages, CD8^+^ T cells and mature DCs, while positively correlating M2-like macrophages in KRASmut LUAD (Fig. 1C).

We next performed immunohistochemistry (IHC) on tumor tissue microarrays (TMA) from 80 KRASmut LUAD patients. High TF-IHC positivity was detected in 53.8% of total cases, which was associated with high TNM stage, metastasis (75%) and disease recurrence (65.51%) (Fig. 1D). In this cohort of KRASmut patients, high TF expression significantly impacted patient survival showing TF-high versus TF-low OS values 39.6 months versus unidentified, and PFS values 20.35 months versus unidentified, respectively (Fig. 1E). To further explore the association among TF and signaling pathway and tumor microenvironment, we performed IHC with representative biomarkers on the same KRASmut LUAD TMA. Results of the combined TNM III + IV cohort (*n* = 29) showed that high TF expression displayed a trend for hyperactive P-ERK and P-AKT signaling and an immune-tolerant tumor microenvironment as shown by an enriched presence of CD206^+^ M2-like macrophage and diminished levels of GZMB^+^CD8^+^ T cells, especially in patients with stages III and IV (Fig. 1F–I, Fig. S2A). These results strongly suggest that high TF expression may reflect a constitutive AKT and ERK signaling and immune-tolerance, both of which likely impact disease progression and poor treatment outcome.

### TF plays a role in KRAS^G12C^ inhibitor resistance and TF inhibition improves therapeutic response

**Fig. 2:**
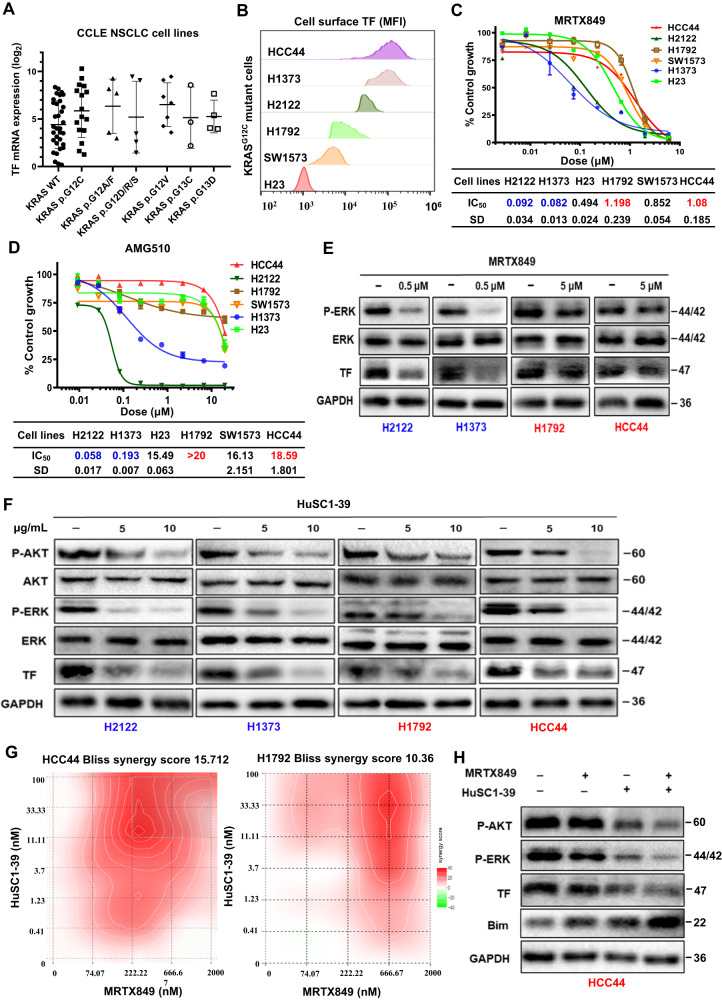
TF-overexpression is associated with resistance to KRAS^G12C^ inhibitors. **A** TF mRNA level in CCLE NSCLC cell lines with KRASwt and various KRASmut genes. **B** Cell surface TF level for 6 KRAS^G12C^ cell lines. **C**, **D** Inhibitor response of 6 KRAS^G12C^ cell lines to MRTX849 (**C**) and AMG510 (**D**). **E** H2122, H1373, H1792 and HCC44 cells were treated with MTRX849 for 72 h, immunoblotted as indicated. **F** Cell lines were treated with HuSC1-39 for 72 h then immunoblotted. **G** Heatmaps of Bliss scores (Synergyfinder software) for HuSC1-39 and MTRX849 combination in HCC44 and H1792 cells. Scores > 10 were considered synergistic, between 10 and -10 were considered additive, < −10 were considered antagonistic. **H** HCC44 cells were treated with 1 μmol/L MTRX849, 10 μg/ml HuSC1-39, or combination for 72 h before immunoblotted. Data are expressed as mean ± SD of 3 technical replicates, representing 3 independent experiments with similar results.

To begin to investigate the potential involvement of TF in disease progression and therapy resistance, we first examined CCLE NSCLC cell line database and found elevated TF mRNA in large percentage of KRASmut NSCLC cell lines (Fig. 2A). Flow cytometry (FACS) detected high TF protein level in a panel of KRAS^G12C^ cell lines (Fig. 2B). We then determined their growth inhibition sensitivity to the clinically approved G12C inhibitors. Among the 6 cell lines tested for MRTX849, H2122 and NCI-H1373 cells were relatively sensitive with their IC_50_ < 100 nmol/L, while H1792, HCC44 and SW1573 cells were defined as relatively resistant cells with IC_50_ values 10-fold higher (Fig. 2C). Among the 6 cell lines tested for AMG510, H2122 and NCI-H1373 cells were similarly shown to be sensitive, while H1792, HCC44 and SW1573 as resistant cells with IC_50_ values 80-fold higher (Fig. 2D). In contrast, KRASi resistance in H23 cells was not associated with TF expression and may be related to Rictor amplification and feedback activation of compensatory signaling pathway [31, 32]. Treatment with 0.5 μM MRTX849 resulted an efficient suppression of P-ERK signaling and a diminished TF expression level in the sensitive H2122 and H1373 cells, while 5 μM MRTX849 treatment had marginal effect in H1792 and HCC44 cells (Fig. 2E). Interestingly, treatment of these same cells with 5 or 10 μg/mL of a TF-blocking antibody HuSC1-39 resulted suppression of both P-ERK and P-AKT pathways as well as inhibition of TF expression, albeit a more potent inhibition was seen in H2122 and H1373 relative to H1792 and HCC44 cells (Fig. 2F). To explore whether a sustained activation of TF contributes to resistance to MRTX849, we tested whether HuSC1-39 could enhance the efficacy of MRTX849. The combination treatment showed concentration-dependent anti-proliferative effect in HCC44 and H1792 cells. Based on the concentration gradients and corresponding inhibition indices, calculated drug synergy scores attributable to drug interactions was 15.712 in HCC44 cells and 10.36 in H1792 cells (Fig. 2G, Fig. S1D). Similarly, the combination of HuSC1-39 with AMG510 also resulted in a synergistic anti-tumor activity (Fig. S1E). Western blotting of HCC44 cells showed that combination treatment caused a more complete inhibition of P-AKT, P-ERK and TF expression compared to either drug alone. Moreover, combination treatment dramatically increased apoptotic tumor cell death as shown by the upregulation of apoptosis-mediated BIM protein (Fig. 2H). These results suggest that TF may mediate KRAS/ERK/AKT function and/or provides a compensatory bypass mechanism in survival and drug resistance.

### TF gene depletion in KRAS^G12C^ inhibitor-resistant cells enhances sensitivity to KRAS^G12C^ inhibition and confers antitumor efficacy in vivo

**Fig. 3:**
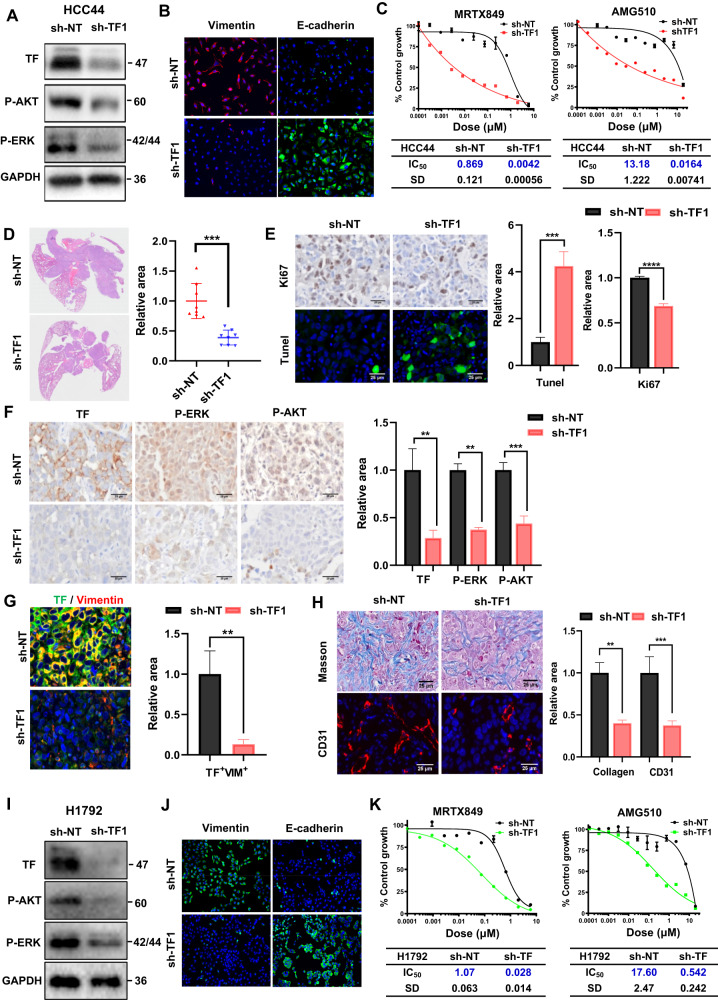
TF-depletion in KRAS-inhibitor resistant cells confers antitumor efficacy. **A**, **B** sh-NT and sh-TF1 HCC44 cells were pre-induced with doxycycline for TF depletion, subjected to immunoblotting (**A**) and immunofluorescence (**B**). **C** Growth inhibition dose response of sh-NT and sh-TF1 HCC44 cells to MRTX849 (left) and AMG510 (right). **D**−**H** shNT and shTF1 HCC44 xenografted tumors were collected, sectioned and stained by H&E dye (**D**). The staining with proliferation marker (Ki67) and apoptosis (TUNEL) (**E**), staining with TF, P-AKT, and P-ERK (**F**), Dual-staining with TF and Vimentin (**G**), and staining with collagen deposition (Masson) and tumor blood vessels (CD31) (**H**) were performed with sh-NT and sh-TF1 HCC44 lung tumor (*n* = 8). **I**, **J** sh-NT and sh-TF1 H1792 cells were pre-induced with doxycycline for TF depletion, subjected to immunoblotting (**I**) and immunofluorescence (**J**). **K** Growth inhibition dose response of sh-NT and sh-TF1 H1792 cells to MRTX849 (left) and AMG510 (right). Data are expressed as mean ± SD of 3 technical replicates, representing 3 independent experiments with similar results, *P < 0.05, **P < 0.01, ***P < 0.001, ****P < 0.0001.

To further determine whether TF plays a functional role in the resistance to KRAS^G12C^ inhibitors, we created TF knockdown HCC44 stable cell clones employing a doxycycline (Dox)-inducible shRNA system. Depletion of TF by two independent shRNA sequences was associated with decreased levels of P-AKT and P-ERK (Fig. 3A, Fig. S3C) and loss of surface expression of vimentin, as well as restoration of E-cadherin expression, the classical markers of EMT (Fig. 3B, Fig. S3D). Importantly, while depletion of TF did not significantly alter HCC44 proliferation rate in culture (Fig. S3B), it greatly sensitized response to KRAS^G12C^ inhibitors MRTX849 and AMG510. The mean IC_50_ values for MRTX849 and AMG510 in TF-depleted cells were reduced by ~200 fold and ~800 fold, respectively (Fig. 3C, Fig. S3E). When implanted orthotopically in the lung, TF-depleted HCC44 tumors grew more slowly, and displayed a significantly reduced Ki67 level and elevated TUNEL staining (Fig. 3D, E, Fig. S3F). Consistent with the in vitro cell culture results, tumors with TF-depletion also downregulated levels of P-ERK and P-AKT (Fig. 3F) and a nearly complete loss of tumor cell vimentin (Fig. 3G). Moreover, we found that TF-depletion reduced tumor angiogenesis and stromal collagen deposition (Fig. 3H, Fig. S3G). In addition, in TF-knockdown H1792 cells, we observed similarly reduced levels of P-AKT and P-ERK (Fig. 3I, Fig. S3I), loss of surface expression of vimentin, and restoration of E-cadherin expression (Fig. 3J, Fig. S3J). Similarly, depletion of TF did not significantly alter the basal proliferation rate of H1792 in culture (Fig. S3H), but greatly increased the response to the KRAS^G12C^ inhibitors MRTX849 and AMG510 (Fig. 3K, Fig. S3K). These results overall indicate that TF overexpression in HCC44 and H1792 cells strongly induces EMT and resistance to KRAS^G12C^ inhibitor, both of which are reversed upon TF-depletion. Targeting of TF can downregulate ERK and AKT signaling, retard tumor growth and improve tumor stroma.

### mTORC2 promotes TF protein stability via proteasome-dependent pathway in KRAS^G12C^ inhibitor-resistant cells

**Fig. 4:**
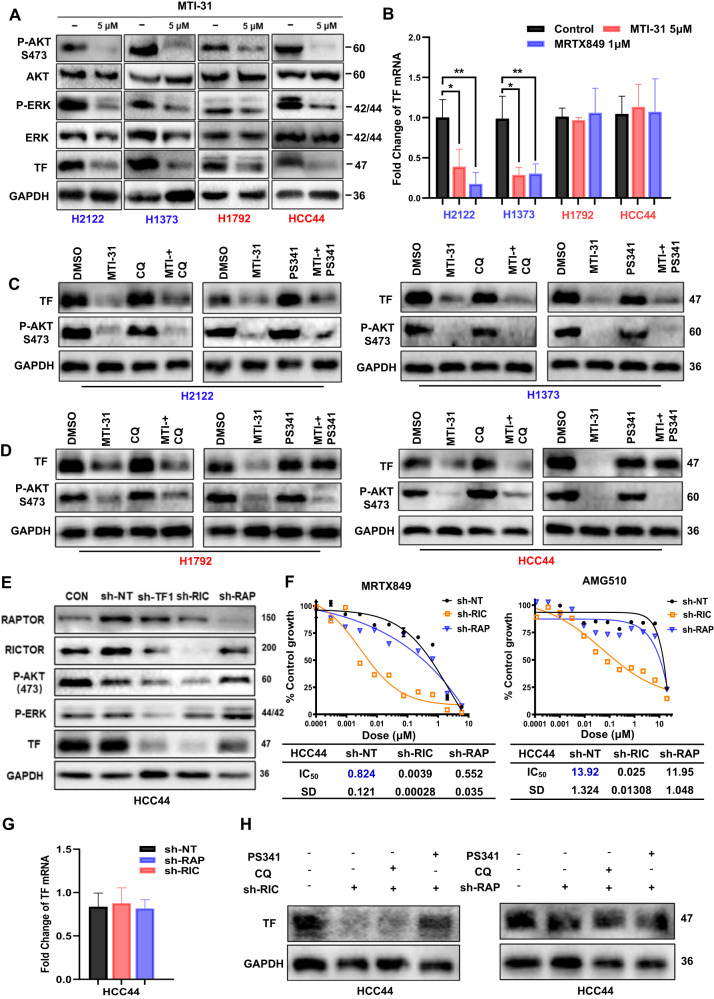
Regulatory mechanism of TF expression. **A** H2122, H1373, H1792 and HCC44 cells were treated with MTI-31 as indicated for 48 h and immunoblotted. **B** The same four cell lines were treated with 5 μM MTI-31 or 1 μM MRTX849 for 48 h and subjected to qRT–PCR analysis for TF mRNA levels. **C**, **D** Cells of H2122 and H1373 (**C**), H1792 and HCC44 (**D**) were treated with 5 μM MTI-31 alone or in combination with 10 nM PS-341 or 10 μM CQ for 48 h, subjected to immunoblotting. **E** sh-NT, sh-TF1, sh-RIC, sh-RAP HCC44 cells were pre-induced with doxycycline for TF depletion then subjected to immunoblotting as indicated. **F** Cells as in (**E**) were treated with titrated doses of MRTX849 (left) or AMG510 (right) for 72 h. Dose response curves are shown. **G** TF mRNA in doxycycline-preinduced sh-RAP and sh-RIC HCC44 cells. **H** Doxycycline-preinduced sh-RAP and sh-RIC HCC44 cells alone or in combination with 10 nM PS-341 or 10 μM CQ for 48 h, subjected to immunoblotting.

Because we observed a significant connection between TF overexpression and AKT signaling in human TMA (Fig. 1G, H) and given the previous evidence for mTOR pathway involvement in KRAS inhibitor resistance [33], we evaluated the effect of mTORC1/2 inhibitor MTI-31 on TF expression. Treatment of H2122, H1373, H1792 and HCC44 cells with 5 μM MTI-31 inhibited mTORC2-substrate P-AKT (S473) and P-ERK in all four cell lines, which correlated with a profound reduction in TF protein suggesting an involvement of mTOR pathway in regulating TF expression (Fig. 4A). qRT-PCR analysis indicated that in the sensitive H2122 and H1373 cells, MTI-31 and MRTX849 each inhibited TF gene transcription, while the identical treatments had no inhibitory effect on TF transcription in the resistant H1792 and HCC44 cells (Fig. 4B). To assess whether mTOR could regulate TF protein stability, the same four cell lines were treated with MTI-31 in the presence or absence of the proteasome inhibitor PS-341 or lysosomal acidification inhibitor chloroquine (CQ). Immunoblotting showed that the MTI-31-induced loss of TF in H2122 and H1373 cells was not influenced by PS-341 or CQ suggesting the inhibition of TF gene transcription as a principal mechanism (Fig. 4B, C). Interestingly, in the resistant HCC44 and H1792 cells, the MTI-31-induced loss of TF was reversed by PS341 but not by CQ implying proteasomal degradation in this process (Fig. 4B, D). To elucidate which mTOR complex(s) is involved in TF regulation, we transfected shRNA depleting Raptor (mTORC1) or Rictor (mTORC2) in HCC44 and H1792 cells. Immunoblotting analysis showed that while depletion of mTORC1 had minor change, depletion of mTORC2 induced degradation of TF (Fig. 4E, Fig. S4A). We then examined growth inhibition dose-response to MRTX849 and AMG510 in HCC44 cells depleted mTORC1 or mTORC2. Depletion of mTORC2, but not mTORC1, greatly sensitized response to MRTX849 and AMG510 with IC_50_ value reduction by ~200 fold and ~500 fold, respectively (Fig. 4F). Moreover, combination treatments with MTI-31 and MRTX849 resulted in a synergistic growth suppression compared to either treatment alone achieving a calculated drug synergy score of 19.146 (Fig. S3A). Importantly, TF mRNA was not altered in mTORC1- and mTORC2-deficient HCC44 and H1792 cells compared to that of sh-NT cells (Fig. 4G, S4B), further confirming that the regulation of TF by mTORC2 occurs at the protein level, whereas TF expression was restored in proteasome inhibitor-treated mTORC2-deficient cells (Fig. 4H, Fig. S4C). Together, these results support a scenario that in KRAS inhibitor-resistant HCC44 and H1792 cells, elevated TF expression is largely due to mTORC2-mediated proteasome-dependent TF protein accumulation. Targeting of mTORC2 can reverse this process and induce TF protein degradation. Like the anti-TF antibody HuSC1-39, targeting of mTORC1/2 by MTI-31 or mTORC2-depletion can also sensitize the therapeutic response to KRAS^G12C^ inhibition.

### Pharmacological targeting of TF or mTOR enhances KRAS^G12C^ inhibitor efficacy in an orthotopic lung tumor model

**Fig. 5:**
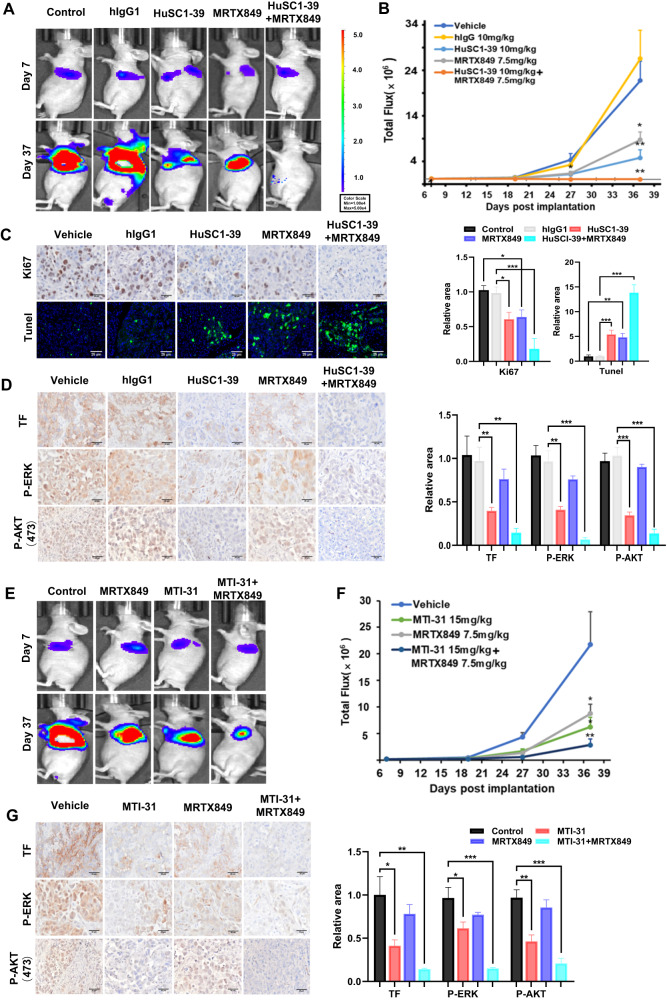
TF/mTOR-blockade enhances the efficacy of KRAS^G12C^ inhibition. **A** Representative bioluminescence imaging of the orthotopic HCC44-luc lung tumor bearing mice used for combination study with HuSC1-39 and MRTX849. Imaging of the full treatment groups (*n* = 6) are shown in Fig. S5A. **B** Tumor growth curves plotted based on bioluminescence “Total Flux” values are shown. **C**, **D** Tumor bearing lungs as in (**B**) were collected and stained with various biomarkers as indicated. **E** Representative imaging of the tumor bearing mice used for combination study with MTI-31 and MRTX849. Imaging of the full treatment groups (*n* = 6) are shown in Fig. S7A. **F** Tumor growth curves are shown. **G** Lung sections from F were stained with various biomarkers as indicated. For all staining images, representative views and relative areas statistics are shown. Error bars represent SEM, *P < 0.05, **P < 0.01, ***P < 0.001.

To assess the therapeutic potential of combination therapy, we first explored combination treatment with HuSC1-39 and MRTX849 in vivo employing an orthotopic lung HCC44 xenograft model. While single agent treatment with MRTX849 or HuSC1-39 each partially reduced tumor growth relative to control, the combination of HuSC1-39 and MRTX849 completely suppressed tumor growth and demonstrated tumor regression in the majority of tumors (Fig. 5A, B, Fig. S5A). We also confirmed this conclusion in the H1792 subcutaneous xenograft model, monotherapy with either MRTX849 or HuSC1-39 partially reduced tumor growth, while the combination treatment with HuSC1-39 and MRTX849 completely inhibited tumor growth (Fig. S6A, S6B). Immunostaining analysis of lung HCC44 tumor sections showed that combination treatment resulted in lower number of Ki67^+^ proliferative cells and more TUNEL^+^ apoptotic cells than MRTX849 or HuSC1-39 alone (Fig. 5C). The combo-treated HCC44 xenografts showed lower collagen deposition and number of CD31^+^ blood vessels (Fig. S5B) and a most complete inhibition of P-AKT, P-ERK and TF (Fig. 5D). Next, we assessed efficacy of combination treatment with MTI-31 and MRTX849 in the same lung orthotopic HCC44 xenograft model. Consistent with the in vitro results, the combo-treatment resulted a further enhanced efficacy compared to either MTI-31 or MRTX849 alone (Fig. 5E, F, Fig. S7A). The combo-treatment also resulted tumor cell proliferation and enhanced apoptosis (Fig. S7B), tumor angiogenesis and collagen deposition (Fig. S7C). Likewise, combination of MTI-31 and MRTX849 led to an efficient downregulation of P-AKT and P-ERK signaling and a complete loss of TF (Fig. 5G). Overall, these results highlight an important role for mTORC2-mediated TF expression in KRAS^G12C^ inhibitor resistance in vivo and suggest a therapeutic potential for use of TF-blocking antibody or mTORC1/2 inhibitor in combination with KRAS inhibitors.

### Combination therapy enhances tumor infiltration of M1-macrophage and TNF-α secretion

**Fig. 6:**
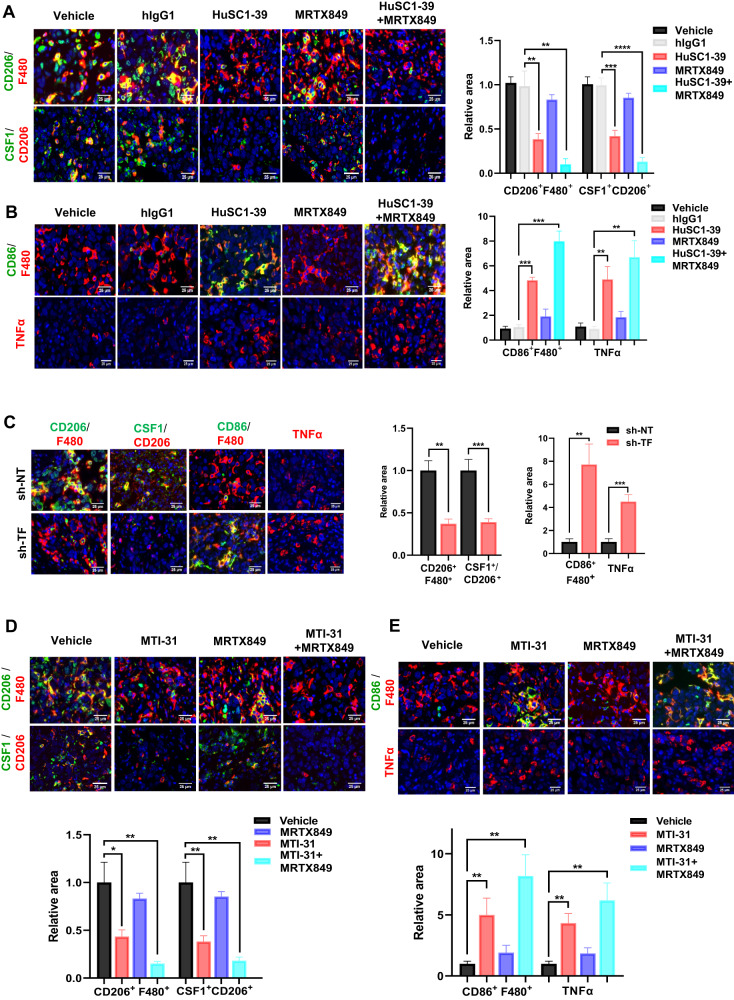
TF/mTOR-blockade enhances M1-macrophage infiltration and function. **A**, **B** Tumor sections generated from Fig. 5B were stained with various biomarkers and analyzed. **C** Tumor sections generated from Fig. 3D were similarly stained and analyzed. **D**, **E** Tumor sections generated from Fig. 5F were similarly stained and analyzed. Representative views and statistical analysis are shown. Error bars represent SEM, **P < 0.01; ***P < 0.001, ***P < 0.0001.

The efficacy of HuSC1-39 or MTI-31 to overcome KRAS inhibitor resistance in vivo is striking and the potential remodeling of tumor microenvironment (TME) may be involved in this setting. Tumor microenvironment is rich in tumor-associated macrophages (TAMs). Most TAMs are tumor-promoting M2-like macrophages, while tumor suppressor M1-like macrophages are the minority. CSF1 is the primary tumor-derived factor responsible for M2 recruitment. We observed that although the total level of macrophages (F4/80^+^) was not substantially affected, the number of M2-macrophages (F4/80^+^CD206^+^) and CSF1 were dramatically reduced and the activated M1-macrophages (F4/80^+^/CD86^+^) were increased in HuSC1-39 or MTI-31 treated tumors (Fig. 6A, B, D, E). This M1-promoting mechanism was also observed in the TF-depleted tumors (Fig. 6C). There were significantly increased amounts of TNF-α secretion in tumors treated with HuSC1-39, MTI-31 or TF-depletion as demonstrated by immunofluorescence (Fig. 6B, C, E). Combination therapy with MRTX849 resulted in a further reduction of intra-tumor M2-macrophages, exhibiting a stronger M1-macrophage phenotype with highest levels of TNF-α (Fig. 6A–E). Taken together, HuSC1-39 and MTI-31 exert their antitumor effects by altering the M2/M1 macrophage ratio and activating M1 macrophage function to overcome KRAS inhibitor resistance. These data further support the importance of the mTORC2-TF axis in disease progression and therapy resistance in KRASmut tumors.

### In vitro model of tumor-macrophage interaction: TF- or mTOR-inhibition facilitates BMDM M1-polarization, phagocytosis and tumor cell death

**Fig. 7:**
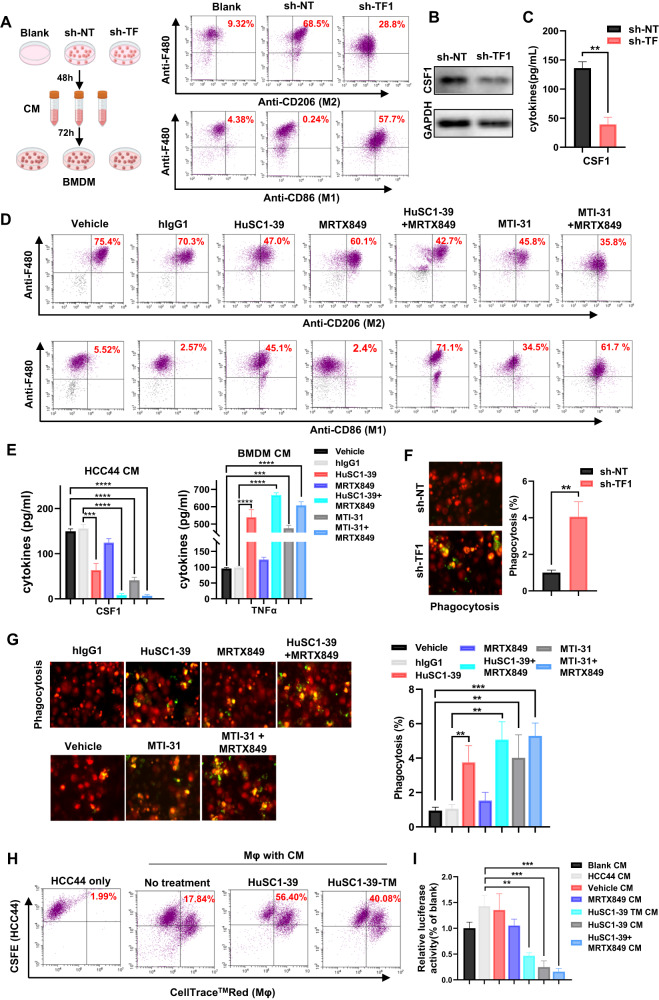
In vitro effects of TF/mTORC2-blockade on TAMs polarization and phagocytosis. **A** As shown in schematic, BMDMs were incubated in sh-NT or sh-TF1 HCC44-CM and subjected to FACS analysis. **B**, **C** sh-NT and sh-TF1 HCC44 lysates were immunoblotted (**B**) or CM subjected to ELISA (**C**). **D** As shown in schematic, BMDMs were incubated with the indicated HCC44-CM and subjected to FACS. **E** CSF1 and TNF-α ELISA were respectively performed on HCC44 CM (Left) or BMDM supernatants after incubated with HCC44-CM (Right). **F**, **G** The CM-incubated and CellTrace^TM^Red-labelled BMDMs were co-cultured with pHrodo™ Green-stained HCC44 cells for 2 h then examined by confocal microscopy. **H** CellTrace^TM^Red-labelled THP-1-derived macrophages were co-cultured with CSFE-labelled HCC44 cells for 2 h, examined for phagocytosis by FACS. **I**. HCC44-luc cells were co-cultured with CM-incubated THP-1-derived macrophage for 24 h. Tumor cell death was measured by luciferase activity. **P < 0.01, ***P < 0.001.

To determine whether targeting of TF or mTORC2 in KRAS inhibitor-resistant tumor cells could influence macrophage polarization in vitro, we performed a cell-based assay where mouse bone marrow-derived macrophages (BMDMs) were incubated with conditioned medium (CM) of HCC44 or H1792 cells. Exposure of BMDM to sh-NT HCC44 or H1792-CM led to increased expression of the M2-marker CD206, promoting macrophage polarization toward the M2-phenotype. Importantly, sh-TF1 HCC44 or H1792-CM suppressed CD206 expression on BMDM and increased its M1-marker CD86 (Fig. 7A, Fig. S4D). Depletion of TF in HCC44 cell decreased synthesis and secretion of CSF1 protein (Fig. 7B, C). In the next set of assays, HCC44 CMs prepared upon blockade of TF/mTOR by HuSC1-39 or MTI-31 reversed M2-polarization and increased M1-marker CD86 expression. The CMs of combined treatment using HuSC1-39 or MTI-31 with MRTX849 synergistically further repolarized macrophages from M2 to M1 (Fig. 7D). The combination therapy further reduced HCC44-derived CSF1 secretion and enhanced TNF-α levels in BMDM (Fig. 7E).

We generated confocal microscopy graphs to visualize phagocytosis. Coculture of CellTrace RED-labelled BMDM and pHrodo™ Green-labelled cancer cell resulted phagocytosis (yellow). BMDM-M2 macrophages treated with sh-TF1 HCC44 or H1792 CM had significantly higher phagocytic rate compared to that of sh-NT group (Fig. 7F, Fig. S4E). Consistently, BMDM-M2 macrophages incubated with HuSC1-39- or MTI-31-treated HCC44-CM engulfed 3- to 4-fold more of KRAS inhibitor-resistant cells than control macrophages, supporting the notion that TF inhibition by HuSC1-39 or MTI-31 promotes macrophage phagocytosis. Combo-treatment with MRTX849 further enhanced phagocytosis of tumor cells by BMDM (Fig. 7G). Next, in the THP-1-derived macrophage phagocytosis assay by FACS, HCC44-CMs of control, HuSC1-39 or HuSC1-39-TM (ADCP deficient) [34] induced phagocytosis rate of 17.86%, 56.4% and 40.08%, respectively (Fig. 7H) indicating that both the ADCP-dependent and independent mechanisms participated. The latter most likely reflected TF-targeted inhibition of mTOR/AKT and ERK pathway function in tumor cells. Lastly, we measured tumor cell death by coculture of HCC44-luc cells with THP-1 macrophages under various CM conditions. While the MRTX849 CM-treated macrophages elicited a limited tumor cell killing as measured by luciferase activity, HuSC1-39 or combination of HuSC1-39 and MRTX849 CM-treated macrophages greatly enhanced the killing effect on HCC44-luc cells compared to control or MRTX849 alone (Fig. 7I). Consistently, the ADCP-deficient HuSC1-39-TM displayed a significant cell killing but not as strong compared to that of HuSC1-39 (Fig. 7I). These results suggest that simultaneous inhibition of TF-mTORC2 and KRAS pathways repolarizes macrophages from a protumor M2 to an antitumor M1 phenotype leading to activation of phagocytic capacity toward cancer cell.

## Discussion

Although the dilemma of mutant KRAS as undruggable target has been overcome, many eligible patients remain nonresponsive to KRAS^G12C^ inhibitors, and acquired drug resistance is likely to develop with standard therapy and KRAS^G12C^ inhibitors [35, 36]. In clinical and preclinical studies, different genomic and histological mechanisms confer resistance to KRAS^G12C^ inhibitors, reactivating ERK signaling bypasses KRAS(G12C) inhibition and drives resistance [37]. Genomic enrichment analysis and mass spectrometry-based phosphoproteomics revealed that induction of EMT promotes resistance to KRAS inhibitors in a cell type-dependent manner through activation of PI3K/AKT or ERK pathways in NSCLC cells and co-suppression ERK or PI3K with KRAS(G12C) may prolong therapeutic benefit [38]. STK11 and KEAP1 mutations commonly co-occur with KRAS mutations in tumors, which contribute to the shaping of immunosuppressive TME, thereby influencing the clinical response to immunotherapy and KRAS^G12C^ inhibitor available in clinical practice [39]. In contrast, the frequently co-mutated gene TP53 is unrelated to treatment outcomes. Our findings reveal a significant upregulation of TF expression in KRAS tumors with STK11 or KEAP1 co-mutations [40]. In the current study, we identified TF as a relevant player in promoting resistance to KRAS^G12C^ inhibition. TF is elevated in high percentage of KRASmut NSCLC and is linked to disease progression, metastasis and patient survival. In our preclinical model, depletion of TF gene or combination treatment with TF-blocking antibody HuSC1-39 each reversed resistance phenotype in vitro and in vivo leading to a robustly enhanced antitumor efficacy toward KRAS^G12C^ tumors. The therapeutic effect of TF-blockade correlated with diminished tumor cell proliferation, increased apoptosis, reduced tumor angiogenesis as well as an overall improved TME. These results, together with emerging clinical experience with single-agent KRAS^G12C^ inhibitors, suggest that combination therapy is necessary for the effective treatment of KRAS^G12C^-mutant NSCLC.

KRAS inhibitor response may critically depend on a sustained inhibition of KRAS-pathway function and sensitivity of KRASmut tumor cells to alternative bypass pathway mechanisms [41, 42]. In the sensitive cells, MRTX849 caused a robust suppression of KRAS downstream P-ERK/P-AKT and TF expression, which, however, was poorly executed in the identically treated resistant cell lines. These results suggest that TF likely confers an alternative bypass mechanism in sustaining the P-ERK/P-AKT activity in the resistant cells. Direct targeting of TF by HuSC1-39 can efficiently downregulate P-ERK/P-AKT activity in both the sensitive and resistant cells, which in a large part explains the restoration of growth inhibition sensitivity to KRAS^G12C^ inhibitors.

We further revealed that TF expression is regulated by two distinct molecular mechanisms in the sensitive versus resistant cells. In the sensitive cells TF accumulation largely relied on the KRAS/P-ERK-dependent TF mRNA transcription, while in the resistant cells TF accumulation involves an mTORC2-dependent suppression of proteasome degradation of TF protein. As we have shown, this mechanism is largely refractory to KRAS^G12C^ inhibitors but can be efficiently targeted by TF-blocking antibody or mTORC1/2 inhibitor, thereby synergizing with KRAS^G12C^ inhibitor in antitumor efficacy. As previous studies have suggested an involvement of mTOR/AKT in KRAS inhibitor resistance [43, 44], our results further propose a notion that mTORC2 may mediate and/or cooperate with TF in orchestrating disease progression and therapy resistance.

Tumor cell EMT is originally recognized as a driver of cancer metastasis. More recent work identified EMT also playing an important role in drug resistance and immune invasion [45–47]. In our drug-resistant cellular and in vivo tumor model, depletion of TF resulted a nearly complete loss of tumor cell surface expression of vimentin, a classical marker of EMT. Our results are consistent with the previous reports on the TF-EMT relationship in triple-negative breast cancer [16, 30] and further suggest that the TF-related EMT may significantly contribute to the drug resistance in KRASmut NSCLC.

TAM is the most abundant immune cell population in TME. M2-TAMs are crucial in tumor immune escape [48, 49]. Our analysis of tumor transcriptome and TMA confirmed a positive correlation between TF and M2 macrophage infiltration in KRASmut patient tumors. In orthotopically implanted lung tumor model, TF expression influenced M2-macrophage polarization. TF-depletion, pharmacological targeting TF or mTORC1/2 in the tumor all enhanced infiltration of M1-macrophage and M1-derived pro-inflammatory activation markers, while diminished M2-related anti-inflammatory markers. These results are recapitulated in a tumor-macrophage interaction in vitro model, where suppression of tumor cell-borne TF or mTORC2 was sufficient to inhibit M2-polarization of BMDM when co-incubated with HCC44-CM. Moreover, we revealed a role for TF/mTORC2 axis in the negative regulation of macrophage phagocytotic capacity resulting in immune invasion and compromised tumor cell killing. This effect can be reversed by TF-depletion or pharmacological targeting of TF/mTOR axis to achieve a significantly improved antitumor efficacy. Mechanistically, the loss of TF/mTORC2 function in tumor cell can reduce CSF1, a critical growth factor of M2-macrophage [50]. Moreover, HuSC1-39 as a TF-targeted hIgG1 mAb can elicit an additional FcγR-mediated immune cytotoxicity to tumor cell.

In summary, our study identified TF as a new and important resistance mechanism of KRAS^G12C^ inhibition. As TF-ADC already demonstrated clinical efficacy in various cancer settings, TF-targeted therapy warrants further investigation for reversing drug resistance in KRASmut NSCLC.

## Materials and methods

### Datasets

The RNA-seq data, survival data and clinicopathological data of 504 lung adenocarcinoma (LUAD) patients were downloaded from TCGA (https://portal.gdc.cancer.gov/). LUAD patients were divided into KRAS mutation group (KRAS-Mut, 155 cases in total) and KRAS wild type group (KRAS-WT, 95 cases in total) according to the KRAS mutation status. Two subpopulations for KRAS mutation group tumors expressing the highest TF (TF-high, *n* = 52) and lowest TF (TF-low, *n* = 52) were subjected to analysis on TF-related prognosis and various biomarkers. Survival analyses were performed in GraphPad Prism 8.0, and logarithmic tests were used to compare statistical differences in overall survival (OS) between the groups. The immune cell fraction of all TCGA samples was analyzed by CIBERSORT (https://cibersortx.stanford.edu/).

KRAS mutated lung adenocarcinoma tissue microarray (TMA) was obtained from Shanghai Outdo Biotech (LD-LUAD1601) containing 80 pairs of tumors and paraneoplastic tissues.

### Immunofluorescence, immunohistochemistry, Masson and TUNEL staining

Paraffin-embedded KRAS mutated lung adenocarcinoma TMA was dewaxed, rehydrated, and subjected to antigen retrieval in 0.01 mol/L citrate buffer (pH 6.0). The slide was blocked with 5% goat serum and incubated overnight at 4 °C with diluted primary antibody. Next day, slide was incubated with a secondary antibody and stained with Tyr-488 and Tyr-CY3 dyes (TSA method) for the immunofluorescence (IF). DAPI was used to stain cell nuclei. The primary antibodies are as follows: TF (E9M6T, CST), P-ERK1/2 (4370, CST), P-AKT (Ser473) (ab81283, Abcam), CD68 (ab955, Abcam), (24595 S, CST), CD8A (50389-T26, Sino Biological) and GZMB (255598, Abcam). Slides were scanned at 20x magnification using a GenePix 4300 microarray scanner. For statistical analysis, immunofluorescence (IF) combined score (CS), quantified as fluorescence intensity multiplied by the percentage of positive cells. ImageJ was used to measure the average fluorescence intensity for each tumor sample. To determine the percentage of positive tumor cells, we selected three different fields (200×) and recorded the percentage of positive cells among all tumor cells in each field. For each staining category, the CS values were converted proportionally to a scalar ranging from 0 to 3, with the highest CS score defined as a value of 3.

Tumors from experimental mouse xenografts were fixed in 4% formalin for 24 h, paraffin-embedded and sectioned. IF, IHC and Masson staining was carried out according to the protocol or method provided by the manufacturer. Primary antibodies used for IHC and IF including TF (E9M6T, CST), P-ERK1/2 (4370, CST), P-AKT (Ser473) (ab81283, Abcam), Ki67 (279653, Abcam), F480(70076 S, CST), CD206 (24595 S, CST), CD86 (19589, CST) and CSF1(ab233387, Abcam). Images were acquired using Leica inverted microscope. Each image (*n* = 3 tumors, 5 image fields of view per section) was quantified using ImageJ software. In total, fifteen representative high-power fields (200×) were selected to quantify the relative area of staining within the tumour tissue. The results of the control group were normalized and then analyzed in GraphPad Prism software.

### Cell lines and immunoblotting

The KRAS mutant human cell lines HCC44, NCI-H23, NCI-H2122, SW1573 and NCI-H1373 were purchased from ATCC. H1792 is a gift from Professor Liu Xiangguo of Shandong University and HCC44-Luc is a gift from Professor Lu Weiyue of Fudan University. HCC44 were infected with luciferase retrovirus (HBLV-luc-BSD, LV57072301) to generate HCC44-Luc cells, and addition of appropriate concentrations of BSD to screen. THP-1 was obtained from Chinese Academy of Sciences. All cells were cultured at 37 °C in a 5% CO_2_ humidified incubator and grown in RPMI 1640 or DMEM medium containing 10% fetal bovine serum and penicillin/ streptomycin (100 U/ml, Life Technologies). All cell lines were tested for mycoplasma contamination.

Protein samples were subjected to SDS-PAGE and transferred to Polyvinylidene difluoride (PVDF) membrane (Millipore, Bedford, MA). The membranes are incubated overnight at 4 °C in the appropriate primary antibody, then washed and incubated with the HRP-conjugated secondary antibody. Primary antibodies were as follows: TF (E9M6T, CST), ERK (4695, CST), P-ERK1/2 (4370, CST), AKT (P31749, Epitomics), P-AKT (Ser473) (ab81283, Abcam), CSF1(ab233387, Abcam), Bim (ab32158, Abcam) and GAPDH (AP0063, Bioworld).

### Cell surface TF levels and gene knockdown

To measure cell surface TF expression, 1 × 10^5^ tumor cells were incubated with serial dilutions of anti-human TF antibody at 4 °C for 1 h, then incubated with R-PE-Goat anti-human IgG-Fc (98596, Abcam). Finally, we used FACS for the assay. TF1-shRNA (V3LHS_371304, Open Biosystem), TF2-shRNA (PGMLV-B2003, Genomeditech), Rictor-shRNA (V2THS_225915, Open Biosystem), Raptor-shRNA (V3LHS_329849, Open Biosystem) or non-targeting -shRNA (NT, RHS4346) lentiviral was packaged in HEK293T cells [30, 51]. Prior to the experiment, tumor cells were stably infected with lentivirus and then induced with 1 μg/mL doxycycline (Dox) for 7 days. The details of shRNA sequence are provided in the supplementary material. The TF1-shRNA sequence is 5’- AAGTCTACACTGTTCAAAT-3’, The TF2-shRNA sequence is 5’-GCGCUUCAGGCACUACAAA-3’.

### qRT-PCR analysis

Total RNA was purified from cultured cells using TRIzol reagent and reverse transcribed using PrimeScript^TM^ RT Master Mix (Takara; RR036A). qRT-PCR was performed according to standard protocols by TB Green® Premix Ex Taq™ (Takara; RR420A). Relative expression of specific genes was normalized to GAPDH levels using the 2-ΔΔCT method. Primer sequences are listed in Supplementary Table S1.

### Flow cytometry, ELISA, macrophage polarization and phagocytosis

Bone marrow-derived macrophage (BMDM) cells from 6-week-old C57BL/6 mice were harvested, and incubated at 37 °C in RPMI-1640 with 10% heat-inactivated FBS and and 10 ng/mL MCSF (Peprotech, Cat# 315-02) for 7 days to differentiate into macrophages. For tumor “education”, different drugs treated HCC44 cells conditioned medium (CM) were collected, and added to bone marrow-derived macrophages (BMDMs) for 48 h, after which expression of M1 and M2 macrophage markers were assayed by flow cytometry.

Commercially available ELISA kit for human CSF1 as well as murine TNF-α (from MULTISCIENCES) were used to detect cytokine secretion from tumor cells and Tumor cell-educated macrophages according to the protocol provided by the manufacturer. The curve of absorbance versus CSF1 and TNF-α concentration in standard wells was generated.

For phagocytosis assay, tumor cell-educated macrophages (BMDM or THP-1) were stained with 200 nM CellTrace-RED for 20 min. Tumor cells were stained with pHrodo™ Green or CSFE at a final concentration of 120 ng/mL for 30 min. After two PBS washes, tumor cells were co-cultured with macrophages for 2 h, examined for phagocytosis using confocal microscopy or flow cytometry.

### Animal models

In vivo efficacy studies were performed under protocols approved by the Institutional Animal Care and Use Committee of Fudan University. To establish a KRAS mutant lung orthotopic transplantation tumor model, luciferase labeled HCC44 cells (1 × 10^6^) were suspended in 50 μL PBS and injected into left lung of nude mice. For tumor staging, mice were injected intraperitoneally with D-luciferin (150 mg/kg) and bioluminescence imaging was performed 6 min later. 7 days after cell injection, tumors were grouped according to fluorescence intensity and randomized into treatment groups (*n* = 6), treated with MRTX849 (7.5 mg/kg orally QD), HuSC1-39 (10 mg/kg i.v. QW), MTI-31 (15 mg/kg orally QD) as the indicated monotherapy or combination therapy. The lung tumor growth was monitored by bioluminescence imaging signals. For H1792 xenograft generation, 5 × 10^6^ H1792 cells were subcutaneously injected into the right flank of nude mice. Once tumors reached an approximate volume of 150 mm^3^, mice were randomly assigned to distinct treatment groups (*n* = 7). Tumor volume was calculated as the formula: length × width^2^ × 0.5.

### Statistical analysis

Numerical data were performed using GraphPad Prism 8 software and Microsoft Excel. Values of in vitro data are mean ± SD, and in vivo values are mean ± SE. *P* values were calculated by an unpaired two-tailed Student *t*-test to determine whether the difference between the two groups was significant. *P* values < 0.05 represented significant statistical difference.

### Supplementary information

supplementary

## Data Availability

All relevant data are within the manuscript and its Additional files.
