# Supplementary material for: Tissue factor overexpression promotes resistance to KRAS-G12C inhibition in non-small cell lung cancer
Source: Oncogene. 2024 Jan 8;43(9):668–81. doi: 10.1038/s41388-023-02924-y (PMC10890931; doi:10.1038/s41388-023-02924-y)
Supplement: Supplementary file 1 — supplementary [file 41388_2023_2924_MOESM1_ESM.docx]

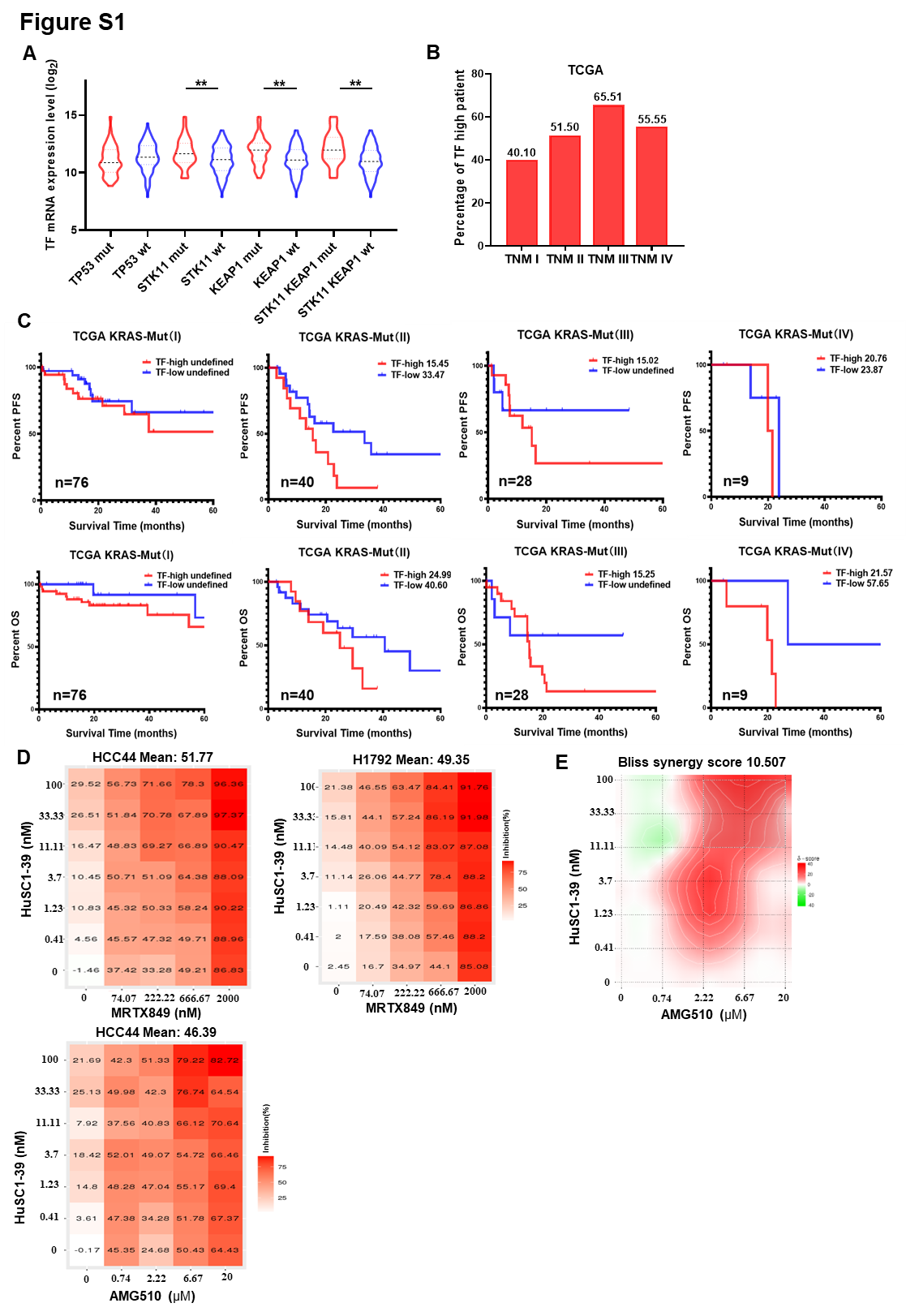


**Figure S1. TF-inhibition synergistically enhances the cytotoxicity of AMG510 against KRAS-inhibitor resistance cells.** **A.** TF mRNA expression level in KRASmut LUAD with TP53, STK11 and KEAP1 co-mutations of TCGA cohort. **B.** Ratio of high TF expression in I, II, III, IV KRASmut LUAD patients of TCGA. **C.** KRASmut patient survival stratified by disease stage (TNM I, II, III, IV) and TF expression in TCGA cohort. Patients without TNM staging were not included in the analysis. **D.** Inhibition rate of HuSC1-39 and MRTX849 combination in HCC44 and H1792 cells. **E.** Heatmaps of Bliss score for HuSCI-39 and AMG510 combination in HCC44 cells, and inhibition rate is indicated numerically.


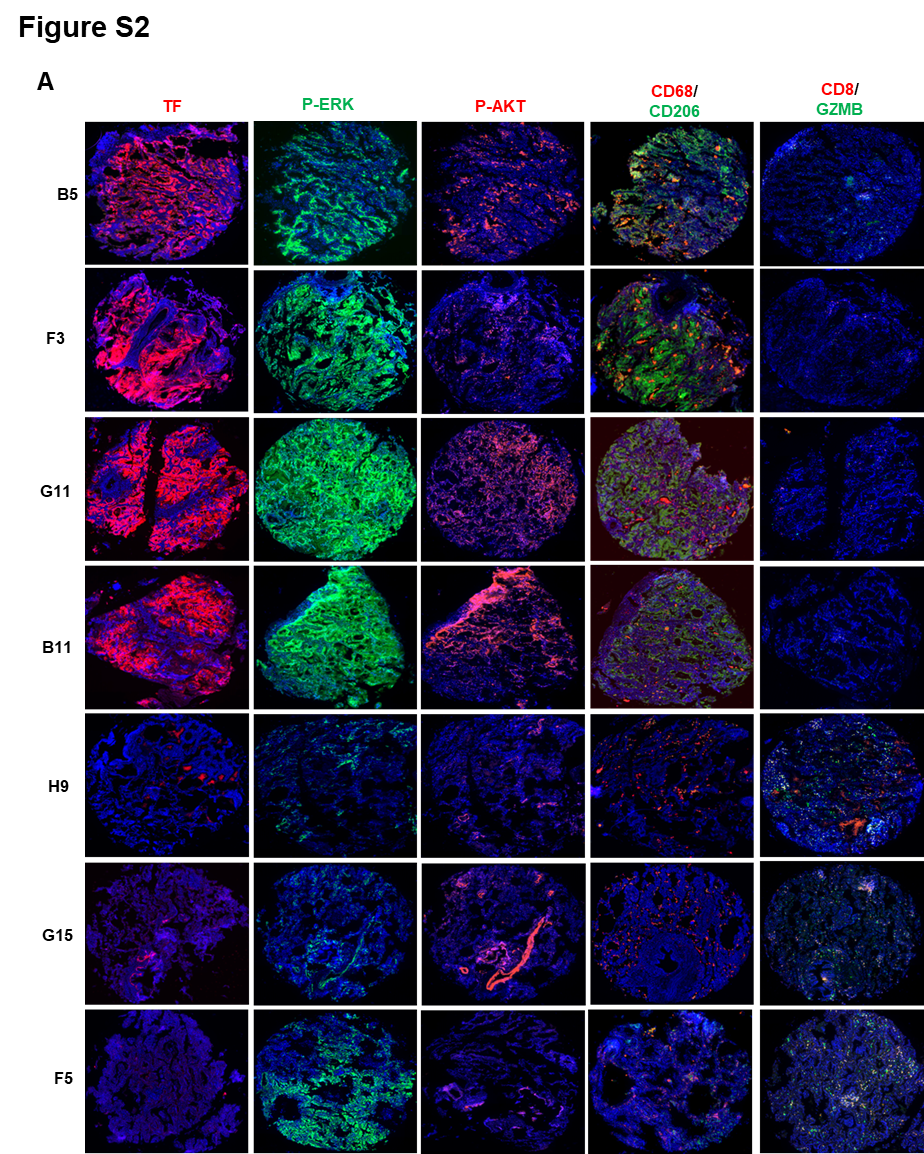


**Figure S2. A.** IHC profile of representative full-slide images was shown in TF high/low KRASmut LUAD patients of stage III and IV.


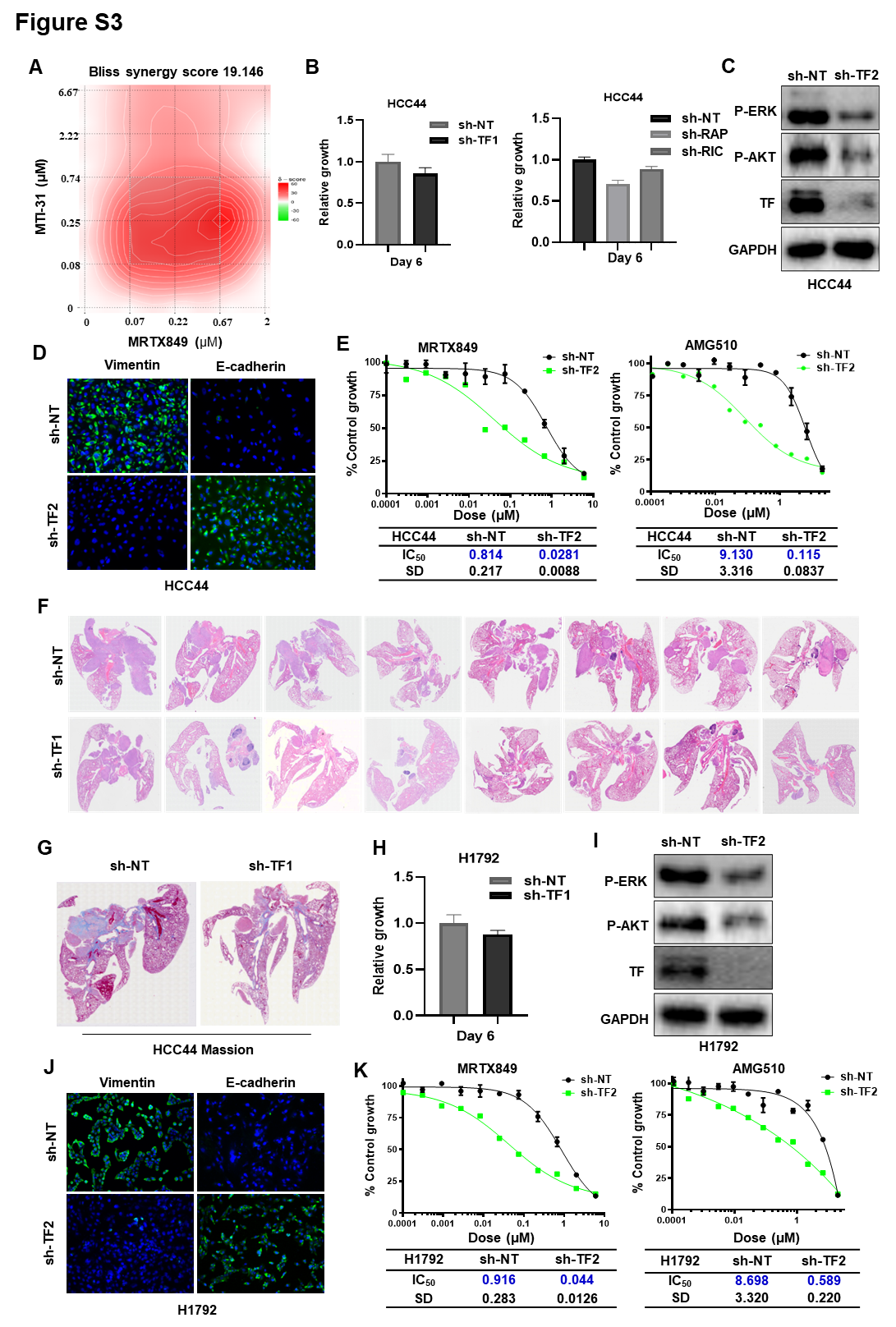


**Figure S3.** **A.** HCC44 cells were subjected to a drug synergy assay with combination matrix doses of MTI-31 and MRTX849. Heatmap of Bliss score for MTRX849-MTI-31 combination treatment response is shown. **B.** Proliferation rate of HCC44 with sh-TF1, sh-RAP and sh-RIC. **C and D.** sh-NT and sh-TF2 HCC44 cells were subjected to immunoblotting (C) and immunofluorescence (D). **E.** Growth inhibition dose response of sh-NT and sh-TF2 HCC44 cells to MRTX849 (left) and AMG510 (right). **F.** shNT and shTF1 HCC44 xenografted tumors were collected, sectioned and stained by H&E dye. **G.** Representative full-slide images of the Masson staining in sh-NT and sh-TF1 HCC44 lung tumor. H. Proliferation rate of H1792 with sh-NT and sh-TF1. **I and J.** sh-NT and sh-TF2 H1792 cells were subjected to immunoblotting (I) and immunofluorescence (G). **K.** Growth inhibition dose response of sh-NT and sh-TF2 H1792 cells to MRTX849 (left) and AMG510 (right).


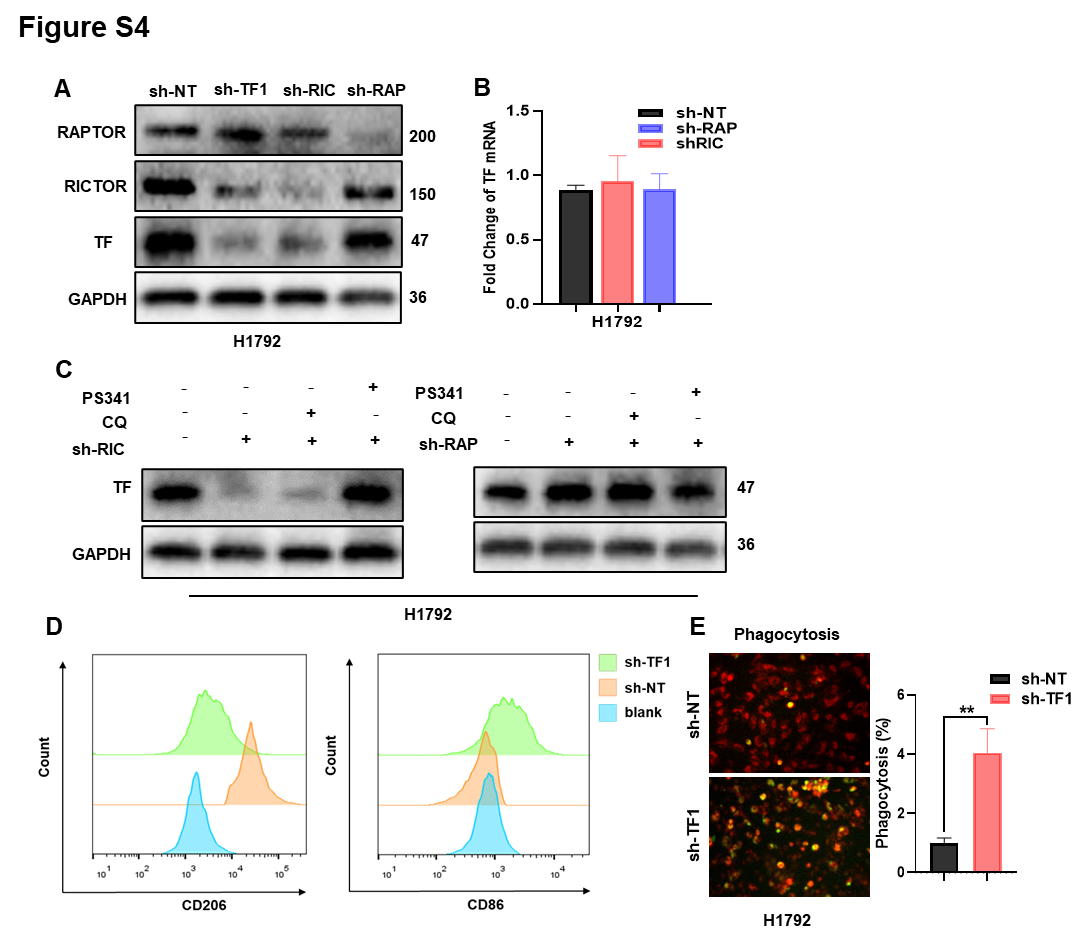


**Figure S4. A.** sh-NT, sh-TF1, sh-RIC, sh-RAP H1792 cells were pre-induced with doxycycline then subjected to immunoblotting. **B.** TF mRNA in doxycycline-pre-induced sh-RAP and sh-RIC H1792 cells. **C.** Doxycycline-pre-induced sh-RAP and sh-RIC H1792 cells alone or in combination with 10 nM PS-341 or 10 μM CQ for 48 hours, subjected to immunoblotting. **D.** BMDMs were incubated in sh-NT or sh-TF1 H1792-CM and subjected to FACS analysis. **E.** Phagocytosis of sh-NT and sh-TF1 H1792-CM cultured BMDM for H1792 cells.


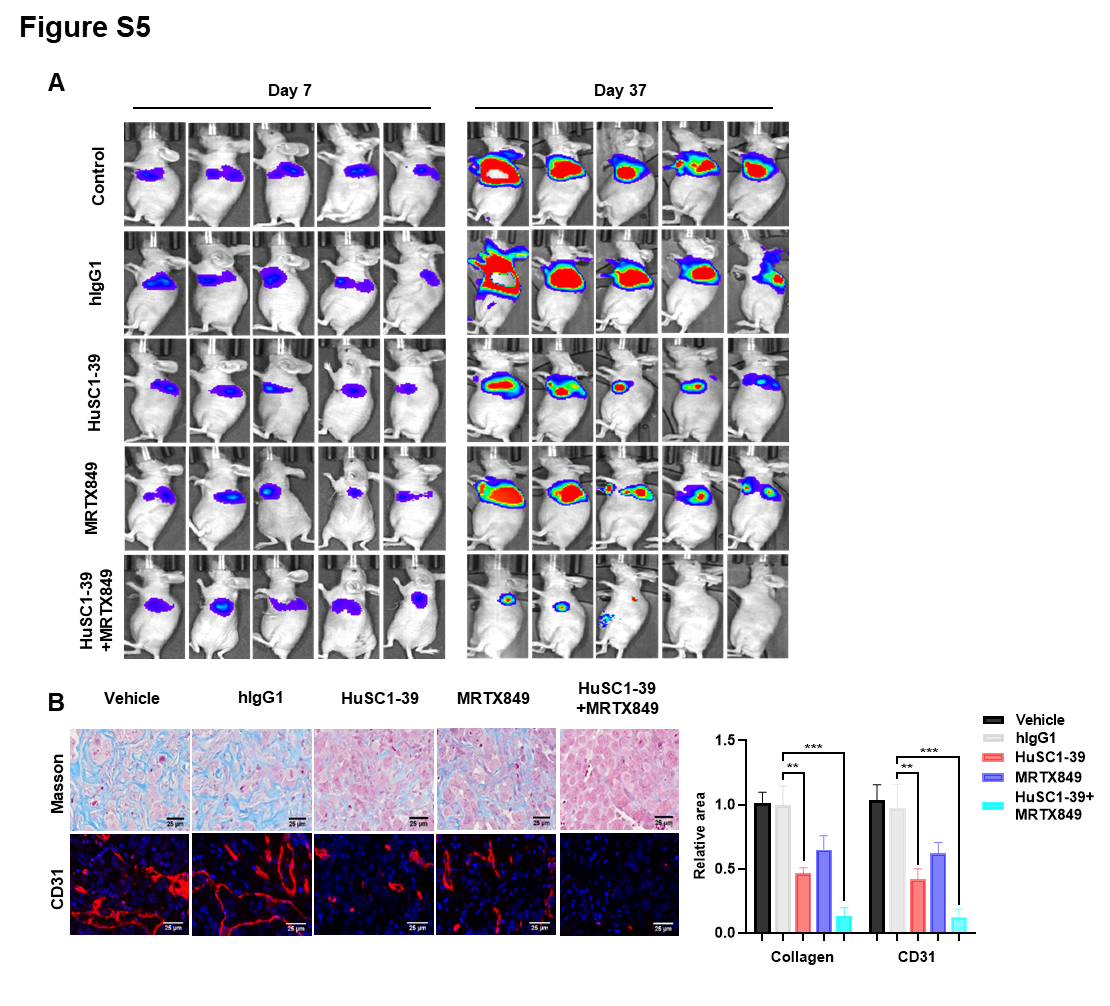


**Figure S5.** **Antitumor efficacy of treatment with MTRX849 and HuSC1-39 alone or combination in KRAS-inhibitor resistant tumor model.** **A.** Bioluminescence imaging of HCC44 xenografts treated with vehicle, hIgG1, HuSC1-39, MTRX849, or the combination (n=6). **B.** Staining of collagen deposition (Masson) and CD31 in HuSC1-39, MTRX849 or combination-treated lung tumors.


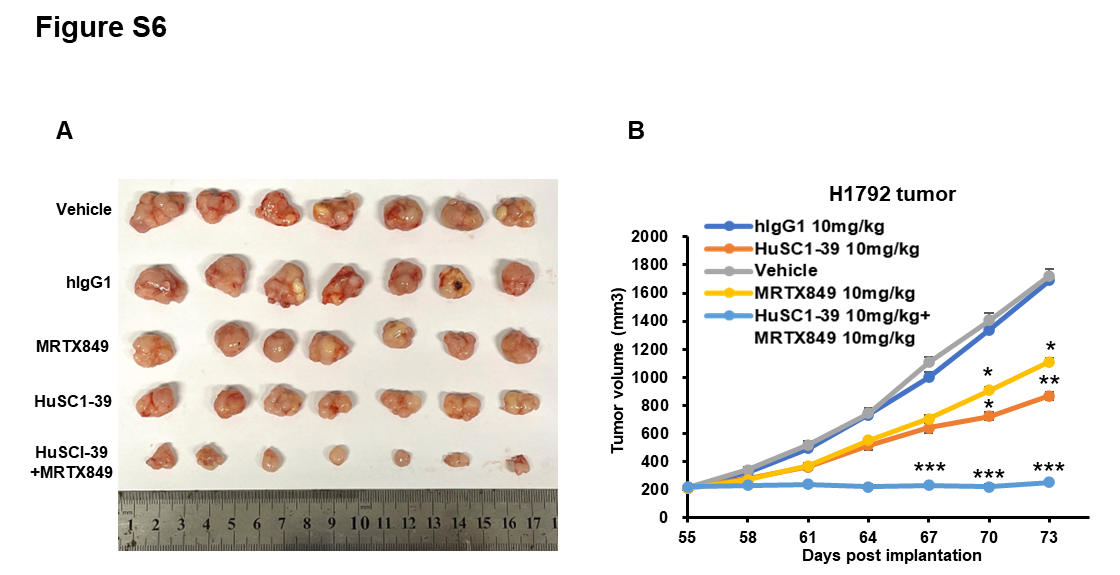


**Figure S6. Antitumor efficacy of treatment with MTRX849 and HuSC1-39 alone or combination in the H1792 subcutaneous xenograft model A.** H1792 tumors treated with 10 mg/kg of HuSC1-39(i.v, QW), MRTX849(orally, QD) or combination. Tumorigenesis was assessed after 73 days as shown. **B.** Tumor growth curves are shown.


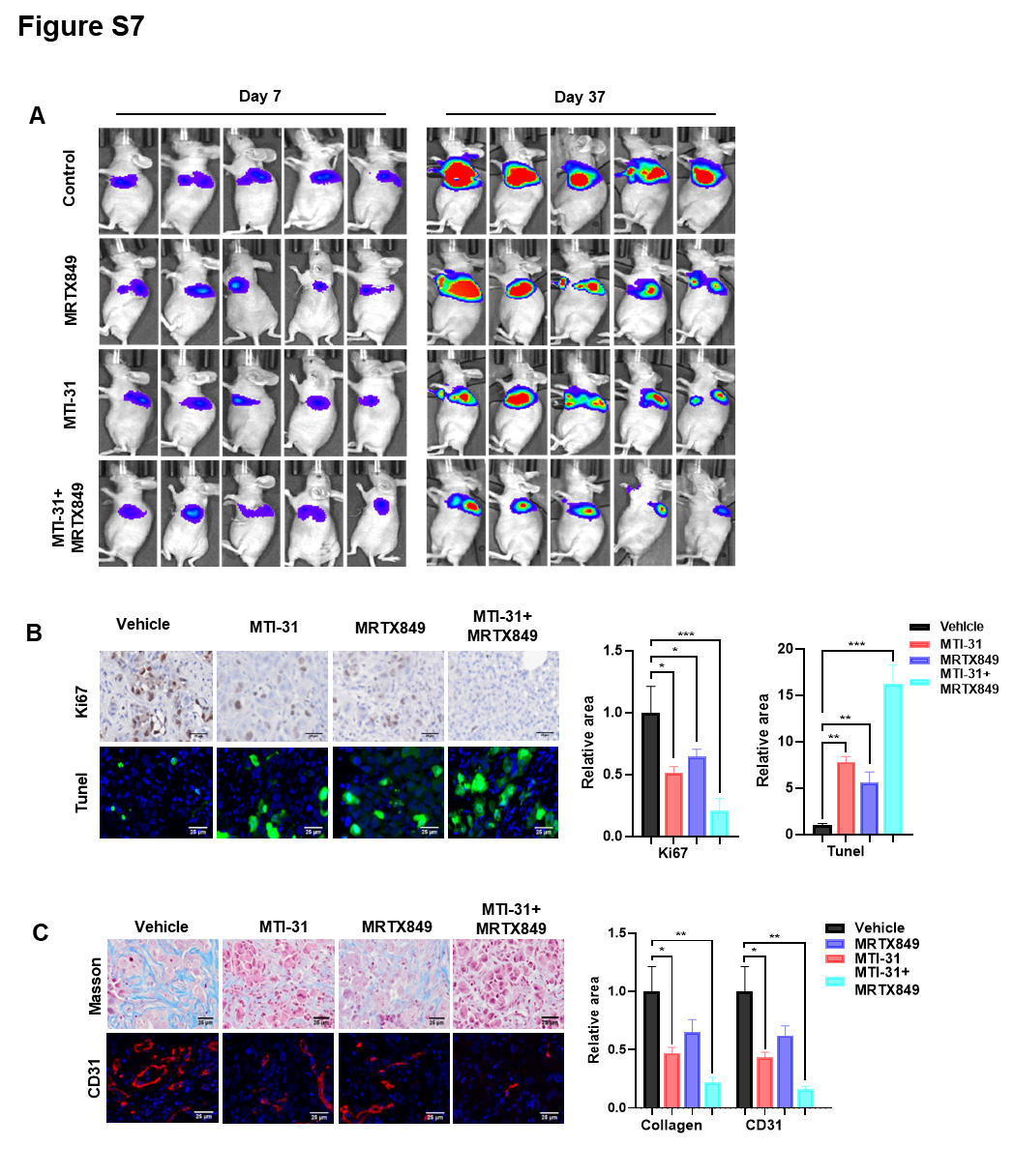


**Figure S7. Antitumor efficacy of treatment with MTRX849 and MTI-31 alone or combination in KRAS inhibitor resistant tumor model. A.** Bioluminescence imaging of HCC44 xenografts treated with vehicle, MTI-31, MTRX849, or the combination (n=6). **B****.** Staining of Ki67^+^ and apoptosis (Tunel). **C.** Collagen deposition (Masson) and CD31 in MTI-31, MTRX848 or combination-treated lung tumors.

**Table S1. primer sequence**

| Gene | Primer sequence forward 5'-3' | Primer sequence reverse 5'-3' |
| --- | --- | --- |
| TF | GGCGCTTCAGGCACTACAA | TTGATTGACGGGTTTGGGTTC |
| GAPDH | GGACTCATGACCACAGTCCA | TCAGCTCAGGGATGACCTTG |
